# Fully Digital Workflow for the Fabrication of Three-Dimensionally Printed Surgical Splints for Preventing Postoperative Bleeding: A Case Report

**DOI:** 10.3390/ijerph191912773

**Published:** 2022-10-06

**Authors:** Masanao Inokoshi, Yumika Soeda, Yo Akiyama, Kaori Ueda, Kazumasa Kubota, Shunsuke Minakuchi

**Affiliations:** Department of Gerodontology and Oral Rehabilitation, Graduate School of Medical and Dental Sciences, Tokyo Medical and Dental University, Tokyo 113-8549, Japan

**Keywords:** postoperative bleeding, 3D printing, intraoral scanner, surgical splint, case report

## Abstract

The management of postoperative bleeding is mandatory in patients receiving anticoagulants. In this case report, we introduce a fully digital workflow for surgical splint fabrication to prevent postoperative bleeding in patients receiving anticoagulants and/or at risk of inadvertent extraction of a mobile tooth during impression making. An 87-year-old woman using apixaban had a left mandibular canine that required extraction due to chronic apical periodontitis. A digital impression was obtained using an intraoral scanner. First, the tooth to be extracted was deleted using three-dimensional (3D) computer-aided design (CAD) software (Geomagic Freeform, 3D Systems) and a stereolithography (STL) file was exported. This modified STL file was imported into another CAD software (3Shape Dental System, 3Shape) and a surgical splint was designed. The splint was fabricated using a 3D printer (Form 3; Formlabs) and light-curable resin (Dental LT Clear, Formlabs) and was delivered after the tooth extraction. The patient was followed-up 2 days after the extraction; no postoperative bleeding was detected and the surgical splint was removed. The additively manufactured surgical splint fabricated using a fully digital workflow was efficacious for managing postoperative bleeding after a dental extraction.

## 1. Introduction

The number of older adults (aged ≥ 65 years) worldwide is increasing, and the population in many countries is a rapidly aging one. Many elderly individuals use medications for systemic diseases, such as cardiovascular and endocrine diseases [1]. Specifically, anticoagulation therapy is frequently used to control atrial fibrillation, valvular heart disease, deep vein thrombosis, and pulmonary embolism. Currently, five oral anticoagulants are predominantly used: apixaban, dabigatran, edoxaban, and rivaroxaban, which are direct oral anticoagulants, and warfarin, which is a vitamin K antagonist [2]. In our previous study published in 2021, the rate of postoperative bleeding after dental extraction was significantly higher with rivaroxaban than with dabigatran [3]. The management of postoperative bleeding is mandatory in patients receiving anticoagulants, and surgical splints should be prepared to properly manage postoperative bleeding.

**Figure 1:**
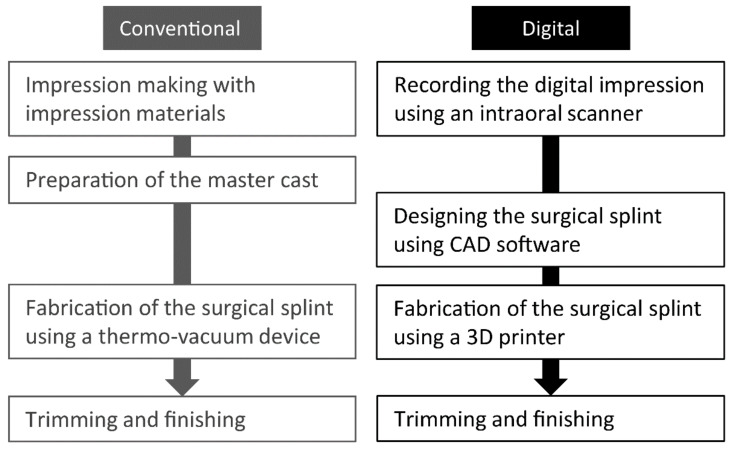
Summary of differences between the conventional and fully digital workflows for fabrication of surgical splints. CAD = computer-aided design.

Conventionally, surgical splints are manually fabricated before the extraction. In the conventional fabrication method, after making an impression and preparing a master cast, surgical splints are fabricated from thermoplastic sheets using a thermo-vacuum device and are trimmed and adjusted prior to insertion in the patient’s mouth. The rapid progress in digital dentistry in recent years has enabled the fabrication of several products in different ways. For instance, fully digital workflows for fixed dental prostheses [4], removable dental prostheses [5,6], and oral appliances [7,8] have been reported. A summary of the differences among fabrication methods of surgical splints is presented in Figure 1. Impression making and the preparation of the master model are replaced by digital impressions using an intraoral scanner (IOS). A modification of the master model can be completed using three-dimensional (3D) computer-aided design (CAD) software. With a fully digital workflow, preparation of master casts is not required, which results in a lower use of consumable materials and a reduction in manufacturing cost [9]. Moreover, the additive manufacturing method allows for the use of a smaller amount of material.

This case report introduces a fully digital workflow for fabricating surgical splints for the prevention of postoperative bleeding in patients receiving anticoagulants, at risk of inadvertent extraction of a mobile tooth, or at risk of aspirating the impression materials during impression making. In this fully digital workflow, a digital impression was obtained using an IOS, and 3D CAD software was used to design a surgical splint, which was fabricated using a 3D printer and light-curable resin.

## 2. Case Report

### 2.1. Ethical Considerations

This report was approved by the ethics review board of Tokyo Medical and Dental University (C2021-010-02).

### 2.2. Patient

**Figure 2:**
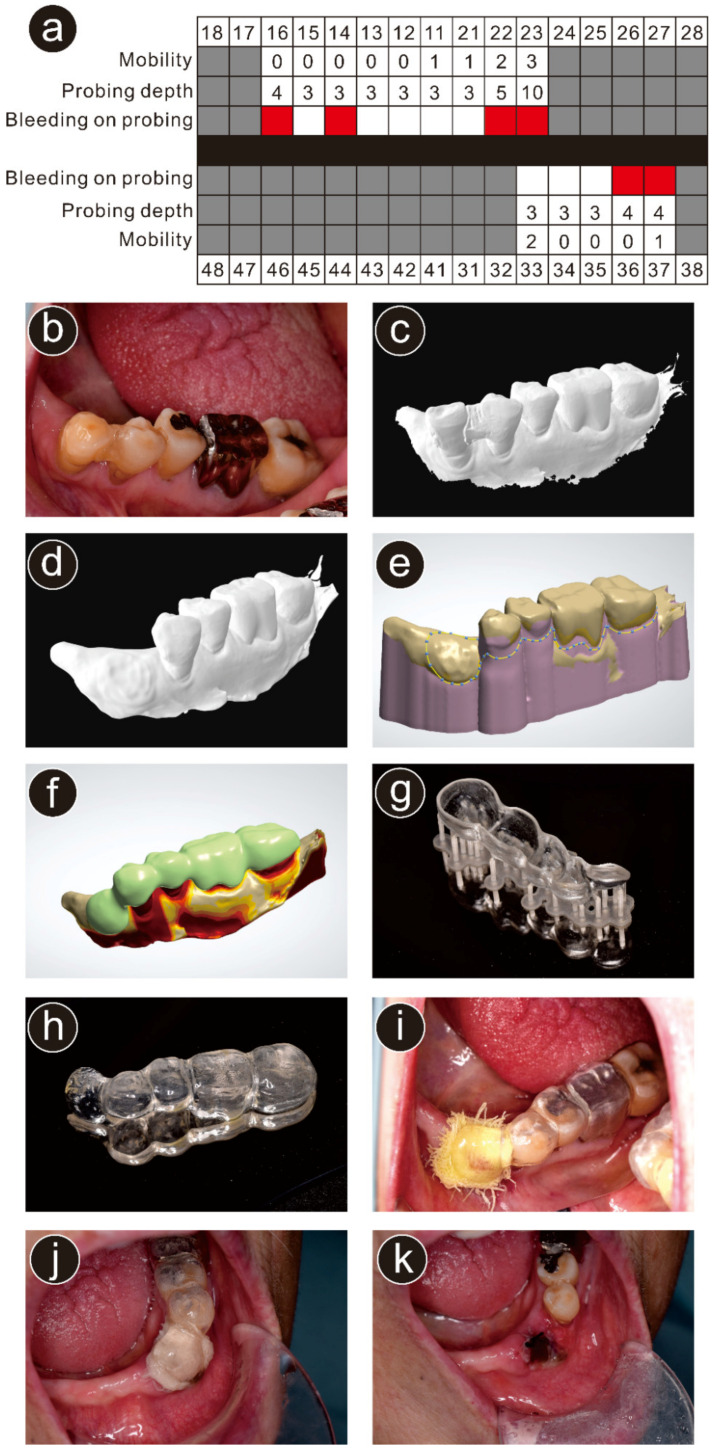
Case presentation of the participant in this report. (**a**) Periodontal chart at the first visit. Gray-colored blocks indicate missing teeth and red-colored blocks indicate bleeding on probing; (**b**) intraoral view before extraction; (**c**) STL data obtained from the intraoral scanner (TRIOS 3); (**d**) stereolithography data after deletion of the left mandibular canine; (**e**) designing the surgical splint using three-dimensional (3D) computer-aided design software; (**f**) designed surgical splint; (**g**) the surgical splint printed using a 3D printer; (**h**) trimmed and finished surgical splint; (**i**) delivered surgical splint; (**j**) intraoral view 2 days after the extraction (before removal of the surgical splint); (**k**) intraoral view of the extraction wound 2 days after the extraction.

An 87-year-old woman with deep vein thrombosis, hypertension, dyslipidemia, anxiety, and insomnia had a left mandibular canine that required extraction due to severe chronic apical periodontitis. The patient’s chief complaint was mobility and pain in the left mandibular canine. The patient was using apixaban for deep vein thrombosis, amlodipine besilate for hypertension, etizolam for anxiety, lemborexant for insomnia, pitavastatin calcium hydrate for dyslipidemia, esomeprazole magnesium hydrate for gastric ulcer, and ezetimibe for dyslipidemia. The patient did not have any specific family and psychosocial history including relevant genetic information. The periodontal chart at the first visit is presented in Figure 2a. A radiographic examination revealed a widened periodontal ligament space. Therefore, the extraction of the left mandibular canine was planned (Figure 2b) after obtaining written informed consent for the procedure and publication of clinical data.

### 2.3. Treatment Procedure

At the first appointment, a digital impression of the mandibular dentition was obtained using an IOS (TRIOS 3; 3Shape, Copenhagen, Denmark; Figure 2c). The acquired stereolithography (STL) data of the patient’s dentition were imported into 3D CAD software (Geomagic Freeform; 3D Systems, Rock Hill, SC, USA). The tooth to be extracted was deleted using 3D CAD software (Geomagic Freeform), and the modified STL file was exported (Figure 2d) into another CAD software (3Shape Dental System; 3Shape) that was used to design a surgical splint (Figure 2e,f). The thickness for most of the surgical splint was set at 1.5 mm with a slightly greater thickness over the extracted tooth (approximately 2.5 mm) to properly press the gauze on the socket.

The STL file containing the surgical splint design was transferred to a 3D printer (Form 3; Formlabs, Somerville, MA, USA), which fabricated a surgical splint using light-curable resin (Dental LT Clear; Formlabs; Figure 2g). After printing, the supporting parts were removed, and the surgical splint was finished (Figure 2h).

The patient was instructed to continue the anticoagulant (apixaban), even on the day of the extraction. After the extraction of the left mandibular canine under local anesthesia using a dental local anesthetic solution of 2% lidocaine with 1:160,000 adrenaline (diluted ORA Inj Dental Cartridget; Showa Chemical Industries, Tokyo, Japan), the surgical splint was inserted into the patient’s mouth (Figure 2i). Postoperatively, the patient was instructed to (1) not remove the surgical splint; (2) not rinse the mouth frequently to avoid postoperative bleeding; and (3) inform the dentist if bleeding was noticed. Two days after the extraction, the patient visited our clinic for a postoperative follow-up examination. No postoperative bleeding was detected (Figure 2j), and the surgical splint was removed (Figure 2k). The patient did not report any complaint regarding the surgical splint which was fabricated using the fully digital workflow.

## 3. Discussion

Surgical splints are fabricated to prevent postoperative bleeding in patients receiving anticoagulants. However, there is a risk of an inadvertent extraction of a mobile tooth or risk of aspiration of impression materials during impression making.

Recently, fully digital workflows for several dental treatments have been developed, including removable dental prostheses [5,6], oral appliances [8,10,11,12], superstructures of dental implants [13,14], and augmentation prostheses for patients with dysphagia [15]. Nishiyama et al. employed an IOS to obtain digital impressions of partially edentulous dentitions and fabricated removable dental prostheses using computer-aided design–computer-aided manufacturing (CAD–CAM) methods [5]. Generally, the enrolled patients were satisfied with their new dentures [5].

Regarding a fully digital workflow for fabricating occlusal appliances, Sohn et al. used digital impressions to design a stabilization splint for the treatment of temporomandibular dysfunction [11]. They concluded that a fully digital workflow for fabricating occlusal appliances could minimize the usage of consumable materials and shorten the operation time. Venezia et al. and Waldecker et al. proposed fully digital fabrication approaches using an IOS and an additive manufacturing method for occlusal appliances [8,12]. They reported that the most significant advantage of applying the CAD–CAM method was data storage and the reproduction of devices with the same shape and thickness.

Joda et al. reported a fully digital workflow of fabricating implant superstructures and concluded that this workflow may be used to develop a feasible treatment approach [13]. Taniguchi et al. employed a fully digital workflow to fabricate implant-supported overdentures (IODs) [14]. They concluded that the fully digital workflow for fabricating IODs is promising because IODs fabricated using the digital workflow allowed masticatory function and patient satisfaction comparable to those with conventionally fabricated IODs. Yoshida et al. reported a fully digital workflow for fabricating palatal and lingual augmentation prostheses [15]. Although the speed and accuracy of the IOS were inferior to those of the conventional technique, they concluded that the treatment outcome was comparable between palatal and lingual augmentation prostheses fabricated using the fully digital workflow and the conventional method. Moreover, they highlighted that employing an IOS has advantages, such as preventing the accidental aspiration of impression materials.

The fully digital workflow of additively manufactured surgical splints has several advantages over the conventional method, such as medical safety, saving time, and saving consumable materials. The most significant advantage of a fully digital workflow is that the dentist can prepare surgical splints even if the patients have other teeth with severe mobility. With the conventional method, severely mobile teeth may be inadvertently extracted during impression making. To avoid an unexpected extraction, dentists are required to make the impression on the day of the extraction and surgical splints need to be prepared during the treatment, which is time-consuming. Moreover, the thickness of the surgical splint cannot be controlled with the conventional method. However, the thickness of a surgical splint fabricated using a 3D printer can be properly controlled, resulting in effective hemostasis after extraction.

Recording digital impressions using IOSs is helpful for the treatment of older adults because it does not involve a risk of aspiration or accidental ingestion of impression materials, especially in patients with Parkinson’s disease or dementia. Thus, the digital impression method is preferred over the conventional impression method in terms of medical safety. Moreover, a fully digital workflow allows the fabrication of other surgical splints from the existing digital impression data without additional impressions. This is advantageous in patients requiring multiple dental extractions. However, IOSs are not always available in dental clinics. Moreover, dental technicians who can handle CAD software and 3D printers are required. These could be some limitations of this approach.

Although dentists can avoid the unexpected extraction of severely mobile teeth during impression making using a fully digital workflow, the size of the IOS may limit its application. Patients must open their mouth widely during digital impression recording.

Although the removal of the supporting parts is required after printing, finishing and trimming are easier for additively fabricated surgical splints than for conventionally fabricated surgical splints, thus reducing the fabrication time of the surgical splint. Patzelt et al. reported that a fully digital workflow of fabricating oral appliances significantly reduced the working time of dental technicians [7]. However, further clinical studies are needed to compare the effectiveness of surgical splints fabricated using the conventional method with those fabricated using the suggested fully digital workflow to prevent postoperative bleeding in patients receiving anticoagulants. Moreover, the cost of fabricating surgical splints may vary across countries. Therefore, future studies should compare the cost effectiveness of the conventional and fully digital workflows.

## 4. Conclusions

A fully digital workflow for additively manufactured surgical splints was introduced. The fully digital workflow of additively manufactured surgical splints has several advantages over the conventional method, such as being less time-consuming and medically safer than the conventional fabrication method, and could be widely applied for patients using anticoagulants and/or at risk of the inadvertent extraction of a mobile tooth or aspiration of impression materials during impression making. The 3D-printed surgical splint fabricated using this workflow could successfully manage postoperative bleeding after a dental extraction.

## Data Availability

The data are not publicly available because they contain information that could compromise the privacy of the patient.
